# The Endless Saga of Monocyte Diversity

**DOI:** 10.3389/fimmu.2019.01786

**Published:** 2019-08-06

**Authors:** Stefania Canè, Stefano Ugel, Rosalinda Trovato, Ilaria Marigo, Francesco De Sanctis, Silvia Sartoris, Vincenzo Bronte

**Affiliations:** ^1^Section of Immunology, Department of Medicine, University of Verona, Verona, Italy; ^2^Veneto Institute of Oncology IOV-IRCCS, Padua, Italy

**Keywords:** monocytes heterogeneity, monocyte continuum, primary tumor, metastatic niche, targeting of monocytes

## Abstract

Cancer immunotherapy relies on either restoring or activating the function of adaptive immune cells, mainly CD8^+^ T lymphocytes. Despite impressive clinical success, cancer immunotherapy remains ineffective in many patients due to the establishment of tumor resistance, largely dependent on the nature of tumor microenvironment. There are several cellular and molecular mechanisms at play, and the goal is to identify those that are clinically significant. Among the hematopoietic-derived cells, monocytes are endowed with high plasticity, responsible for their pro- and anti-tumoral function. Indeed, monocytes are involved in several cancer-associated processes such as immune-tolerance, metastatic spread, neoangiogenesis, and chemotherapy resistance; on the other hand, by presenting cancer-associated antigens, they can also promote and sustain anti-tumoral T cell response. Recently, by high throughput technologies, new findings have revealed previously underappreciated, profound transcriptional, epigenetic, and metabolic differences among monocyte subsets, which complement and expand our knowledge on the monocyte ontogeny, recruitment during steady state, and emergency hematopoiesis, as seen in cancer. The subdivision into discrete monocytes subsets, both in mice and humans, appears an oversimplification, whereas continuum subsets development is best for depicting the real condition. In this review, we examine the evidences sustaining the existence of a monocyte heterogeneity along with functional activities, at the primary tumor and at the metastatic niche. In particular, we describe how tumor-derived soluble factors and cell-cell contact reprogram monocyte function. Finally, we point out the role of monocytes in preparing and shaping the metastatic niche and describe relevant targetable molecules altering monocyte activities. We think that exploiting monocyte complexity can help identifying key pathways important for the treatment of cancer and several conditions where these cells are involved.

## Introduction

Monocyte diversity is well-recognized but the biologic and clinical significance of the different monocyte subtypes is far from being completely elucidated. The main hallmark of monocytes is their plastic nature, whereby they can exert multiple roles during the course of the immune response including cytokine production, pathogen clearance, antigen presentation, wound healing, and pro/anti-tumoral response (1–3). The original classification of monocytes into classical (in humans: CD14^high^, CD16^−^; in mice: Ly6C^high^), intermediate (in humans: CD14^high^, CD16^low^), and non-classical (in humans CD14^low^, CD16^high^; in mice: Ly6C^low^) is currently being replaced by evidences supporting the existence of a “monocyte continuum” rather than stepwise differences between the different subtypes (4). Indeed, in mice under steady state, circulating classical monocyte subsets have been shown to switch into non-classical monocytes over time (5–7). However, it remains to be shown what relationship exists among the human monocytic subsets, and whether and how pathological conditions, like inflammation and cancer, impact this process.

Circulating monocytes have been viewed for many years, as precursor cells that provide tissue macrophages and dendritic cell (DCs) populations (8, 9); however, mounting evidence suggests that monocytes have their own effector functions in the blood and at peripheral sites throughout the body (10). The emerging data that distinct monocyte subsets, carrying different genetic, epigenetic, transcriptional, and metabolic arrangements, are committed to become macrophages and DCs seems to contradict the general accepted view of monocytes responding to a particular environmental stimuli and then differentiating into multifaceted macrophages and DCs. The intriguing evidence, both in mice and humans (11–13), of trained-monocytes, both present as mature and precursor cells, seems to strongly support the former hypothesis and reinvigorate the idea that monocytes have specific functions beyond being precursor cells. In this review, by combining the most recent advances in the field of monocytes' genetic, epigenetic, transcriptomic, and metabolomic, we outline and evaluate the changes occurring in monocyte subsets that underlie the aforementioned plasticity and heterogeneity. Secondly, we discuss new concepts in the monocyte field, like trained immunity and reprogramming and highlight the targetable pathways controlling monocyte fate and function.

We think that combining the information of single-cell transcriptome profiling, metabolomics array and epigenetic studies will elucidate complex relationships between cell types, thus solving limitations in the existing classification that relies on a relatively small number of markers.

## Monocyte Phenotypical and Transcriptional Plasticity

### Inflammatory and Patrolling Monocytes

Studies over the past two decades have delineated two major subsets of monocytes in mice and humans. Inflammatory monocytes (iMo), characterized by the high expression of the chemokine receptor CCR2, are repeatedly released from the bone marrow into the circulation. These cells, alternatively known as classical monocytes, are Ly6C^hi^ in mice and correspond to the CD14^hi^CD16^lo^ monocyte subset in humans. The fate of these cells is strictly dependent on the state of the body. Under steady state conditions, extravasated iMo and their derived-cells enrich in nearly all tissues throughout the body, where they form a small yet significant group of the so called local tissue-resident macrophages (7). The gradual accumulation of monocyte-derived macrophages in tissues is generally associated with the slow but progressive replacement of embryonic macrophages, in both quantitative and qualitative fashion (14). Monocyte-derived macrophages present sustained gene modifications as compared to their circulating counterparts, as they shape to the tissue microenvironment. They acquire transcriptional signatures resembling resident macrophages of embryonic origin, even though a certain level of differences remains at the epigenetic, transcriptional and functional levels (15–17). Whether monocyte-derived macrophages that infiltrate tissues under steady-state condition gain a self-renewal ability, comparable to their embryonic counterparts, is still a matter of debate and seems to strictly depend on the type of infiltrated tissue. On the other hand, iMo can also conserve their monocyte-like state inside the tissues without differentiating into macrophages, thereby acting as a local monocyte reservoir (18) (Table 1).

**Table 1:** Summary of monocyte subsets presented in this review, highlighting their markers and function in both humans (top part of the table) and mice (bottom part of the table).

**Monocyte subset**	**Markers of identification**	**Function**
**HUMAN**		
Inflammatory monocytes (iMo)	CCR2^+^/CD14^high^/CD16^low/neg^	Inflammatory response
Patrolling monocytes (pMo)	CX3CR1^+^/CD16^high^/CD14^low^	Tissue repair
Immunosuppressive monocytes (M-MDSC)	CD11b^+^/CD14^+^/CD124^+^/PD-L1^+^/CCR2^+^/HLA-DR^−^/ARG1/IDO1/cFLIP/IL-6/IL-10/TGFβ/STAT3/cEPBβ/NF-κB	Immune dysfunction, tumor angiogenesis and vasculogenesis, promotion of metastasis, promotion of tumor cell stemness
Trained monocytes	CD14^+^/Dectin1^+^/CD36^+^/TLR4^+^/GM-CSFR^+^/NOD/mTOR/ERK1/ERK2/NLPR3/HIF1α/aerobic glycolysis/TNFα/IL-6/IL-1β/H3K18Ac/H3K4me/H3K27ac	Innate immune memory that balance the equilibrium of balance of immune homeostasis, priming, training, and tolerance
SatM-expressing monocytes	Undefined	Not yet identified in humans
Neutrophil-like monocytes	Undefined	Not yet identified in humans
**MOUSE**		
Inflammatory monocytes (iMo)	SSC^int^/CD11b^+^/F4/80^+^/CD64^+^/Ly6C^high^/CD43^low^/CD62L ^+^/CD115^+^/CCR2^+^/CX3CR1^−^/MHCII^low/−^/IRF8/KLF4	Inflammatory response
Patrolling monocytes (pMo)	SSC^int^/CD11b^+^/F4/80^+^/CD64^+^/Ly6C^low^/CD43 ^high^/CD62L^−^/CD115^+^/CCR2^−^/CX3CR1^+^/MHCII^low^/TREML4	Tissue repair
Immunosuppressive monocytes (M-MDSC)	CD11b^+^/Ly6C^+^/Ly6G^low/neg^/CD124^+^/PD-L1^+^/CCR2^+^/ARG1/NOS2/cFLIP/IL-6/IL-10/TGFβ/STAT3/STAT1/STAT6/cEPBβ/NF-κB/Chop/S100A8/S100A9	Immune dysfunction, tumor angiogenesis and vasculogenesis, promotion of metastasis, promotion of tumor cell stemness
Trained monocytes	Ly6C^low^/Dectin1^+^/CD36^+^/TLR4^+^/GM-CSFR^+^/NOD/mTOR/ERK1/ERK2/NLPR3/HIF1α/aerobic glycolysis/lactate/mevalonate/TNFα/IL-6/IL-1β/H3K18Ac/H3K4me/H3K27ac	Innate immune memory that balance the equilibrium of balance of immune homeostasis, priming, training, and tolerance
SatM-expressing monocytes	Ly6C^low^/Flt3^−^/FcεR1^+^/CEACAM1^+^/F4/80^−^/Mac1^+^/C5aR^+^/M-CSFR^+^/MSR1^+^/cEPBβ/MPO- and NE-containing granules	Fibrosis
Neutrophil-like monocytes	Ly6C^+^/MPO- and NE-containing granules	Response to microbial components (i.e., LPS) and maintaining homeostasis at steady-state

Besides the aforementioned pathway of maturation, iMo can either remain in the blood, or transition into patrolling monocytes (pMo) by the setting up of *de novo* enhancers and activation of “frosted” enhancers (19, 20) (Table 1). The mechanisms driving the conversion of iMo into pMo are just beginning to be elucidated. It appears that Delta-like 1 (Dll1) signal from endothelial cells by interacting with NOTCH2 only iMo favors their switch into pMo cells (21). These data clearly indicate that iMo and pMo monocytes are biologically intertwined, corroborating observation obtained at the epigenetic level, which indicated that both monocyte subsets use the same promoter repertoire and minimally differ in their chromatin organization (19). Of course, this scenario raises several questions: are the iMo infiltrating the tissues able to reprogram into pMo? Is this switch tissue dependent? Can we interfere with this reprogramming to control the transition? Is there any factor maintaining iMo reservoir? Are pMo thus originated able to re-enter the blood stream? Are monocyte-derived macrophages transcriptionally similar to pMo? In mice, iMo can give rise to pMo, even though this does not rule out the presence of an alternative route for pMo development, independent from the iMo subset (8). Indeed, genetic evidence for this transition do exist. Two myeloid-determining transcription factors, like interferon regulatory factor 8 (IRF8), and the downstream Kruppel-like factor (KLF4), have been shown to regulate iMo generation without affecting the pMo numbers. Moreover, studies conducted on either global IRF8^−/−^mice, or fetal liver transplant of KLF4^−/−^cells into irradiated wild type mice, indicate a drastic reduced numbers of iMo in the bone marrow, while maintaining relatively normal pMo numbers (22). These findings suggest a pathway for pMo development untied from the iMo subset, probably originating directly from the common monocyte progenitor (cMoP). The identification of the transcription factors nerve growth factor IB (NR4A1) has helped to withstand the hypothesis that pMo can arise independently from iMo monocytes. On the other hand, recent single-cell RNA sequencing of murine and human monocytes indicate that circulating iMo and pMo represent, under physiological conditions, a nearly homogenous populations (19). Interestingly, data recently published (23) combining single profile and functional and phenotypic characterization, showed that monocytes subsets (defined as classical, intermediate, and non-classical) isolated from peripheral blood of both healthy mice and humans, can be further divided into two additional populations: one group expressing classical monocyte genes and also cytotoxic genes and the other one with undefined activity. Other studies conducted in human and mouse lung cancer samples (24) have showed that several tumor-infiltrating myeloid populations (TIM) and among those monocytes are uniquely associated with the disease and with clinical progress, highlighting the potential to use TIM as immunotherapeutic targets. We think that the multiple cell subsets identified in the aforementioned manuscripts, should be tested for their functional relevance in tumor progression, in disease progression and their abundance should be correlated with therapeutic response. Of course, the correlation between human and mouse TIM will help to achieve these goals with the ultimate purpose of gaining more insight into monocytes and monocyte-dependent therapies.

In contrast, patrolling monocytes (pMo) represent a more differentiated subset and are marked by the higher surface expression of CX3CR1. pMo express low levels of Ly6C in mice and are CD14^lo^CD16^hi^ in humans; they routinely check the vessels under physiological conditions through the engagement of an LFA/ICAM-dependent crawling mechanism with resting endothelial cells (25, 26). This patrolling behavior of pMo can be observed throughout the interdigitated system of capillaries, arterioles, and venules. Similarly, human CD14^lo^ CD16^hi^ pMo show patrolling behavior when adoptively transferred into immuno-compromised mice (27). The crawling features of pMo allows them to efficiently sense particles, on the one hand, and on the other hand to monitor of endothelial cell integrity. However, these cells are not restricted to the vessels as pMo also undergo diapedesis and can be identified within the parenchyma of multiple tissues (7). pMo display a longer lifespan at the steady state compared to iMo and they are, also for this reason, found in the blood, at any given time, more abundant than their counterpart (5, 28). Interestingly, pMo cells are strongly susceptible of the physiological status of the organism and therefore they might represent a potential diagnostic tool (29). Nevertheless, how the fine balance between iMo and pMo levels and differentiation capacity is maintained/regulated during severe inflammation, autoimmune diseases, and cancer is just began to be elucidated.

**Figure 1:**
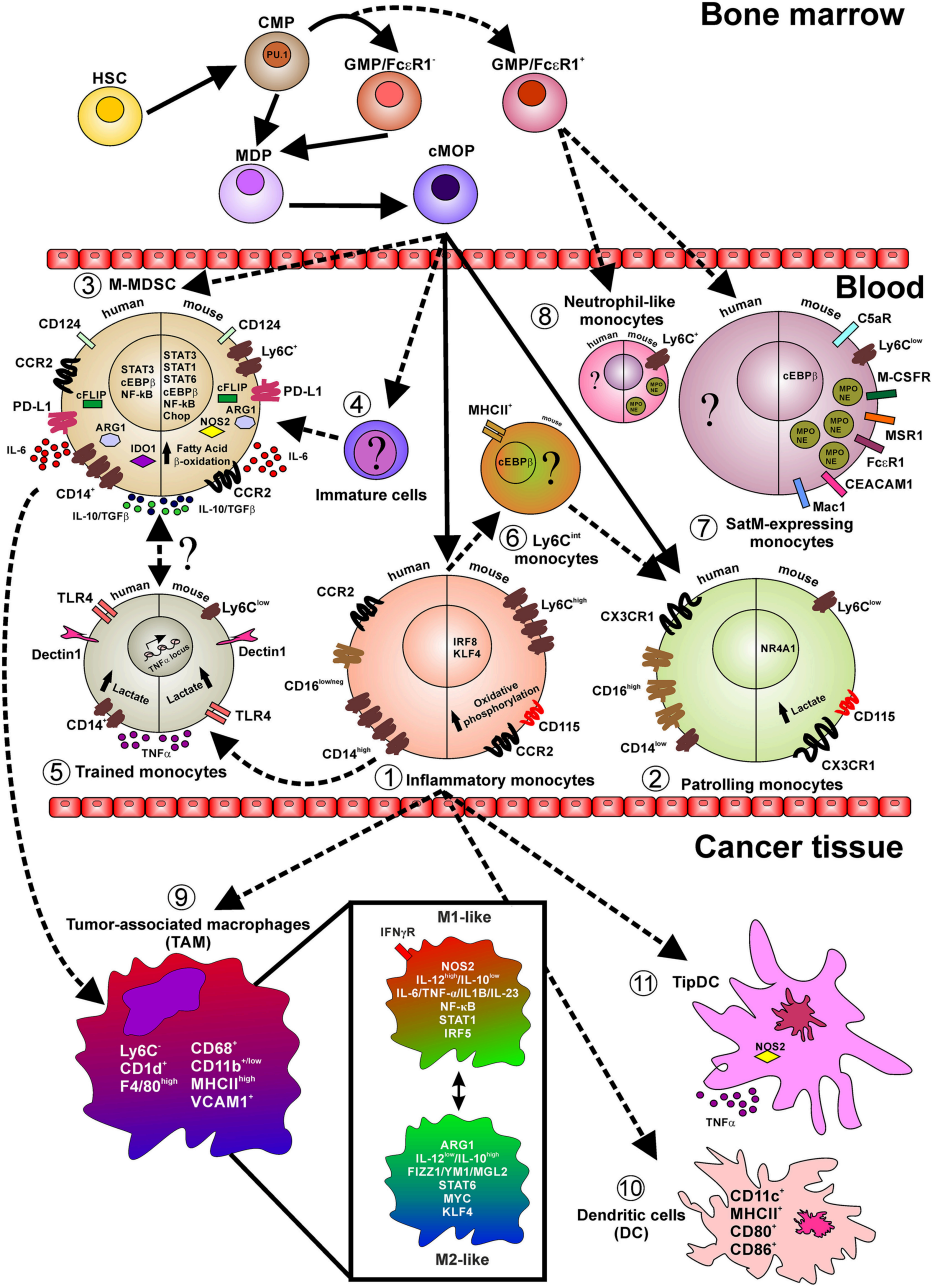
Layout depicting the monocyte lineage precursors (on top), the monocyte subsets in the peripheral blood (center), and monocyte fate in cancer tissues. Indicated are relevant surface markers, transcription factors, secreted cytokines, intracellular mediators, and relevant metabolic pathways. Continuous lines indicate events occurring during normal myelopoiesis while shaded lines indicate events in emergency myelopiesis (e.g., cancer and inflammation). Briefly, under steady state cMop precursors originate both inflammatory (1) and patrolling (2) monocytes, both in humans and mice. However, it has been reported that during emergency myelopoiesis cMop precursors can also differentiate into M-MDSC (3) and into not yet defined immature cells (4). Particularly, during infection, inflammatory monocytes acquire a trained phenotype (5) and also switch into Ly6C^int^ cells (6) only identified in mice and with not fully defined function, transcriptional regulators, and markers. During fibrosis a novel subset of monocytes, so called SatM (8), have been characterized, in mice, defined as Ly6C^+^ and expressing proteins typical of the neutrophil granules (MPO and NE). These cells, together with neutrophil-like monocytes (7), found in peripheral blood of mice during microbial infection and in the bone morrow in steady-state condition, originate from GMP/FcεRI^+^ precursors cells in the bone marrow. In pathological conditions, like cancer, inflammatory monocytes infiltrating the tissue give rise to TAM (9) which in turn represent a fultifaced population of macrophages. Additionally, inflammatory monocytes can also differentiate into classical DC (10) expressing the costimulatory molecules CD80 and CD86 and TipDC (11) expressing high level of NOS2 and TNFα.

Under pathological conditions, such inflammation and cancer, the rapid recruitment of myeloid cells to sites of injury stimulates a constant development and mobilization of cells from the bone marrow, causing a state of “emergency” that might generate monocytes from different ways or precursors. These include monocytes that have circumvented the canonical MDP-cMoP-monocyte developmental axes and resemble neutrophil-like iMo derived from GMP precursors (30). An additional example of a recently described monocyte subset that appears under inflammatory conditions is the segregated nucleus-containing atypical pMo (SatM) (31). SatM and neutrophil-like monocytes represent a minor pool of monocyte subsets under steady-state (19, 30, 31) (Table 1), yet become conspicuous during inflammation (30, 31). At present, the lack of reliable surface markers, associated with a deep epigenetic and transcriptional profile unable to make a clear distinguish between neutrophil-like iMo identified using GFI1/IRF8-reporter mice (30) from classical iMo and SatM (identified as Ly6C^low^Ceacam1^hi^F4/80^−^Mac1^hi^), from non-classical pMo (31). The limited whole-cell proteomic data available so far showed, for example, that SatM cells contain granules expressing granulocyte-related protein, like myeloperoxidase (MPO) and neutrophil elastase (NE) (31) (Figure 1). These cells are related to fibrosic responses; in fact, adoptive transfer of SatM monocytes into bleomycin-treated mice exacerbates fibrosis. Furthermore, it was shown that chimeric mice, lacking the CCAAT/enhancer-binding protein beta (Cebpb) in the hematopoietic progenitors were resistant to fibrosis when exposed to bleomycin but they had unaltered inflammatory response, further supporting the role of SatM in sustaining the mechanism of fibrosis (31). Nevertheless, more studies will be required to uncover the origin of these subsets and their involvement in different pathologies. To add more complexity, a recent work by Hanna et al. (32), showed that a subset of circulating pMo, but not iMo, accumulate at the site of tumor where they display an anti-tumoral role, by directly engulfing cancer cells and by releasing factors which in turn activate cytotoxic natural killer cells (NK). Are these extravasating pMo similar to their blood counterpart? How the findings from Hanna et al. (32) correlate with observations that extravasating pMo can differentiate into macrophages during cancer? Are pMo “corrupted” by tumor cells once they have extravasated within tissues and switched to pro-tumoral cells? Are iMo and pMo competing for the access to the tumor site? Are iMo suppressing the anti-tumor function of pMo? Indeed, this mechanism is also consistent with the data from the pMo adoptive transfer experiments since pMo appear to act early during seeding but not after establishment of metastatic foci despite their continued accumulation. Therefore, under pathological conditions it still remains possible that pMo derive from either blood iMo, via the formation of an intermediate Ly6C^int^ monocyte, or from bone marrow Ly6C^+^ monocytes, from an independent bone marrow monocyte progenitor, or from a combination of all these pathways (Figure 1). In order to solve all these issues a detailed understanding of the factors and pathways regulating the development and survival of both iMo and pMo populations in specific inflammatory settings is necessary.

As mentioned before, adoptive transfer and fate-mapping studies support the hypothesis that monocytes develop along a differentiation continuum in which inflammatory monocytes give rise to the patrolling subset in the circulation (3, 5). Development of monocytes from bone marrow progenitors combines the regulated expression of numerous transcription factors, with the contribution of growth factors and cytokines (33). Monocytes and neutrophils are both derived from hematopoietic stem cells (HSCs) via common myeloid progenitors (CMPs), which can originate from granulocyte-monocyte progenitor (GMP) and monocytes/macrophages, DC precursors (MDPs) (34, 35) (Figure 1). The commitment toward monocytes is characterized by three major lineage-determining factors (LDTF): PU.1, CCAAT/enhancer-binding protein beta (Cebpb) and Cebpa. PU.1 is a master regulator of myeloid and lymphoid cell development (36). The genetic ablation in mice of PU.1 promotes a lethal embryonic phenotype and the transfer of PU.1 mutated stem cells favors an altered myelopoiesis characterized by a robust contraction in both monocytes and DCs (37, 38). By binding closed chromatin through its C-terminal DNA-binding domain (39), PU.1 acts as a coordinator for the activation of selected genomic regions in collaboration with monocyte-associated transcriptional factors such as the IRF8 and KLF4 (40). Moreover, PU.1 synergistically cooperates with C/EBP-δ to activate the promoters of interleukin-6 (IL-6) and CCL5 (41) and transactivates the human macrophage colony-stimulating factor receptor (M-CSFR) promoter via the c-Jun pathway (42). Proteins of the C/EBP family radically impact the myeloid cells development. Since in *Cebpa*-deficient mice the transition from CMPs to GMPs is completely abrogated, these mice lack the granulocytic compartment (43, 44) indicating that C/EBPα is the master regulator of steady-state granulopoiesis.

Additionally, during myelopoiesis, C/EBPα controls and activates the myeloid-associated gene expression program by binding to either promoters or enhancers of myeloid-related genes, such as colony stimulating factor 3 receptor (CSF3R), growth factor independent-1 (GFI-1), interleukin 6 receptor (IL-6R), or C/EBPε, both in mice and in human stem cells (45). C/EBPα inhibits specific transcriptional factors attenuating the expression of non-myeloid lineage genes (46, 47). Moreover, genetically enforced inducible C/EBPα expression in GMPs by tamoxifen administration favors monocyte development demonstrating the critical role of this transcriptional factor during monopoiesis (48). By contrast, C/EBPβ is not necessary for steady-state granulopoiesis. However, C/EBPβ was recently identified as the key factor during the epigenetic default differentiation of iMo monocytes into pMo cells under steady state condition, highlighting the multifaceted role of this transcriptional factor during monocyte development (19). In this regard, a key contribution of C/EBPβ to mylopoiesis is highlighted by data showing that mice knockout for *Cebpb* have a dramatic reduction in circulating monocytes (19). C/EBPβ is also the master regulator of emergency myelopoiesis; in fact, inflammatory signals (i.e., cytokine stimulation) strongly induce the downregulation of all members of C/EBP family except for C/EBPβ (43). Under pathological condition, like fibrosis, a C/EBPβ-associated gene program in FcεR1^+^ GMPs progenitors promote the development of alternative monocytes, like SatM, previously described. As we will discuss below, C/EBPβ-driven programs are also activated in cancer-educated myeloid cells.

### Myeloid-Derived Suppressor Cells

In cancer, tumor-derived soluble factors, such as growth factors, cytokines, chemokines, and tumor-derived exosomes, not only support an increased recruitment of monocytes from bone marrow to tumor-microenvironment bypassing the canonical monocyte development but, also, favor the acquisition of immunosuppressive features in myeloid cells. To highlight these acquired functional properties, myeloid cells comprising monocytes, neutrophils, and immature cells were named myeloid-derived suppressor cells (MDSCs) (49, 50). Although this terminology generated some controversies, it represents a useful ground for scientific researches on altered hematopoiesis. It relies on the concept that myelopoiesis in pathology might give rise to cellular subsets that can share some markers with the cells present under steady state but are functionally and molecularly distinct, sharing the property of negatively regulating effectors of adaptive and innate immunity. The monocytic-MDSC (M-MDSC) subset is broadly defined in mice as Ly6C^+^CD11b^+^ cells and in human as CD14^+^HLA-DR^−^ or CD14^+^CD124^+^ cells and is endowed with a stronger ability to arrest T cell response, when compared to the granulocytic-MDSC (G-MDSC) counterpart (51, 52), in part dependent on the activation of two enzymes, arginase 1 (ARG1) and inducible nitric oxide synthases (NOS2/iNOS), which are directly regulated by C/EBPβ expression (53) (Figure 1; Table 1). We demonstrated that, in tumor-bearing mice, both the expansion and the immunosuppressive function of MDSCs are abrogated in the absence of C/EBPβ, resulting in restricted tumor spread (53). These data confirm the central role of C/EBPβ in tumor-associated inflammation, underscoring it as promising therapeutic target to develop new approach to limit cancer progression. Both human and mouse M-MDSCs secrete immunoregulatory cytokines, like IL-10, TGFβ, and IL-6 and present and array of molecules, such as ARG1, FADD-like IL-1β-converting enzyme-inhibitory protein (c-FLIP), indoleamine 2,3-dioxygenase 1 (IDO1), and nitric oxide synthase 2 (NOS2), which can contribute to the suppressive activity of these cells. Mechanistically, IL-6, for example, activates PI3Kγ, which stimulates mTOR, S6Kα, and C/EBPβ-mediated anti-inflammatory gene expression and inhibits NFkB-mediated pro-inflammatory gene expression, thereby promoting the immune suppressive function of these cells mediated by, but not limited to, IL-10, TGFβ, and ARG1 (54, 55). In particular, *Arg1* gene in MDSCs is strictly controlled by several inducible transcriptional factors able to recognize sequences characterized by high content in GC that impacts the nucleosomal stability (56), such as signal transducer and activator of transcription 3 (STAT3), IRF8, as well as CHOP, PU.1, KLF4, and activator-protein 1 (AP-1) (57). Moreover, STAT3 promotes both expansion and survival of M-MDSCs through Bcl-XL, c-Myc, and Cyclin D1 expression (58) as well as the induction of several immune regulatory mediators like bFGF, HGF, VEGF, IL-1β, MMP9, CCL2, and CXCL2 (50). Interestingly, phosphorylated STAT3 binds to multiple sites in the *Arg1* promoter, suggesting that STAT3 inhibitors, like Stattic, could reduce ARG1 dependent immunosuppression by dampening the expression of *Arg1* mRNA (59). Within the tumor environment, ARG1 can cooperate with NOS2 to produce high levels of superoxide anion (${\text{O}}_{2}^{-}$) that can react with either nitric oxide (NO) or H_2_O generating reactive–nitrogen species (RNS), such as peroxinitrites (ONOO^−^), which damage both the function and migration of T cells to tumor site (60), and reactive-oxygen species (ROS), such as H_2_O_2_ which decreases T cellular CD3ζ expression limiting the activation of T cells, respectively (61). However, ARG1 has a hierarchically dominant negative role compared to NOS2 in developing an immunosuppressive tumor microenvironment by limiting the activity of monocyte-derived NOS2-expressing and TNF-producing dendritic cells (defined as Tip-DCs) that can sustain and favor the anti-tumor effect of transferred T lymphocytes (62). An alternative way to reprogram MDSC differentiation and function is through the expression of p53. It was recently demonstrated that M-MDSC can be driven to differentiate into potent antigen-presenting, defined as Ly6C^+^CD103^+^ DCs by inflammation-induced activation of p53. In fact, mice with a targeted deletion of p53 in myeloid cells specifically loose the Ly6C^+^CD103^+^ population and became unresponsive to different forms of immunotherapy and immunogenic chemotherapy (63).

Recently, we demonstrated the ability of c-FLIP, which controls the extrinsic apoptotic pathway and caspase 8 activation (64), to re-program monocytes into MDSC-like cells (65). In fact, FLIP-expressing monocytes displayed impressive regulatory features both *in vitro*, constraining the activated T cell proliferation, and *in vivo*, controlling the development of graft vs. host disease in a xenogeneic mouse model. Indeed, enforced expression of c-FLIP in monocytes up-regulates MDSC-associated immunosuppressive genes, such as CD273, CD124, IL-6, IL-10, CFS3, PTGS2, and IDO1, as a result of a “steered” NF-kB activation induced by the nuclear co-localization of c-FLIP with NF-kB p50 (65). During the course of a disease, like cancer, MDSCs infiltrate the tumor, differentiating into tumor-associated macrophages (TAMs) which can sustain primary tumor growth and contribute to the pre-metastatic niche formation (Figure 1).

### Trained Immunity and Metabolic Landscape of Monocyte Subsets

Recent studies have shown that during infection with some pathogens iMo can undergo extensive epigenetic, transcriptional, and metabolic reprogramming, with the functional consequence of an enhanced immune reactivity upon a second encounter, in other words they acquire an immunological memory. The existence of this innate immune memory was initially suggested by studies in mice deficient for functional T and B cells and exposed to mild *C. albicans* infection, which show protection against *C. albicans* reinfection by increased responsiveness of monocytes (66). Even though the requirements for monocyte training has been primarily investigated either *in vitro* or under *in vivo* steady state, trained monocytes seem to originate from iMo during emergency hematopoiesis by a profound epigenetic and metabolic rewiring (Table 1). Exposure of iMo to either *C. albicans* or β-glucan *in vitro*, induce profound genome-wide changes in epigenetic marks, including, but not restricted, histone H3 lysine 4 monomethylation (H3K4me1), trimethylation (H3K4me3), and H3 lysine 27 acetylation (H3K27ac) (67) as a consequence of Dectin-1/AKT/mTOR/HIF-1α signaling pathway activation and secretion of IL-6, IL-1β, and TNFα. Other studies instead, identified Bacille Calmette-Guérin (BCG) and peptidoglycan as potent inducers of the aforementioned trained-related epigenetic modifications, though a different mechanism dependent on nucleotide-binding oligomerization domain-containing protein 2 (NOD2) pathway and activation of NF-kB (68). Concomitantly to these epigenetic changes, a metabolic switch also occurs. Trained monocytes are mainly glycolytic (aerobic glycolysis) with impairment of the oxidative phosphorylation, production of lactate and disruption of the Krebs cycle at the level of both citrate, which is withdrawn for fatty acid biosynthesis, and succinate, which activates HIF-1α and consequently up regulates the expression of several pro-inflammatory cytokines, mainly IL-1β and TNF-α (69).

Beside microbial particles, products of the lipid metabolism were found to be activators of the trained immunity. Oxidized-low density lipoprotein (oxLDL), a damage-associated molecular pattern (DAMP), interacts with CD36 on myeloid cells leading to the activation of NLRP3 inflammasome and consequent production of IL-1β (70–72).

Although the role of epigenetic programing as a mechanism required to insure innate immune memory is becoming more clear, one crucial aspect still remains unanswered: what is the cellular process that induces and maintains such epigenetic changes? Initial evidences seem to suggest that metabolites might play a role since they can act as cofactors for the enzymes (mainly methylases, methyltransferases, histone deacetylases, and histone acetylases) involved in epigenetic modulation of gene transcription (67, 73, 74). Of course, more studies are required to fill the gap and also to deeply examine the role that different chromatin modifications have on the stability of the chromatin. It is expected that stable histone modifications (e.g., histone methylation) would be more suitable to maintain a functional modification than those with short half-life (e.g., histone acetylation). Thus, the long-lasting persistence of some histone modifications could reflect both the stability of such modifications or the persistent activation status of the signaling pathways and transcription factors upstream (75, 76). Understanding these regulations *is a sine qua non* for designing therapeutic intervention aimed at modulating trained immunity, to dampen it when in excess (e.g., organ rejection, autoimmunity, allergy, atherosclerosis) or enhance it when defective (e.g., cancer, infection).

A growing body of evidence suggests that the development of immune cells and their different effector functions are the results of a dynamic changes occurring at the metabolic level (77, 78). In mouse models of cancer, myeloid cells are metabolically influenced by tumor-derived factors to become MDSCs, helping to protect tumor from the effects of chemotherapy (79). Specifically, mouse MDSCs undergo a major metabolic reprogramming by switching off glycolysis and enhancing fatty acid β oxidation (FAO) pathway. This metabolic reprogramming is generally characterized by an up-regulation of lipid uptake receptors CD36 and Mrs1, an increase in FAO enzymes, mainly carnitine palmitoyltransferase 1 (CPT1) and 3-hydroxyacyl-Coa dehydrogenase (HADHA), and an increase in oxygen consumption. These events are associated with the activation of immunosuppressive pathways, namely upregulation of ARG1 and NOS2 synthesis and production of ONOO^−^, contributing to dampen T cell proliferation and IFNγ secretion (80–82). Blockade of FAO, both *in vitro* and *in vivo* in different tumor models, decreased the incorporation of fatty acid and ATP production, holding up the development of suppressive MDSCs (82) and leading to increased efficacy of chemotherapy and adoptive T cell therapy. Interestingly, fatty acid oxidation also plays an important role in regulating the inflammatory properties of iMo. Increased intracellular level of unsaturated fatty acid (arachidonic acid) was shown to stimulate the secretion of pro-inflammatory IL-1α by uncoupling the mitochondrial respiration (83, 84) thus exacerbating the pathogenesis of atherosclerosis (85). The relevance of changes in the lipid metabolism occurring during myeloid cells differentiation, was also recently demonstrated by Mitroulis et al., in *an vivo* model of trained immunity (13). Treatment with β-glucan determines an increase in gene expression of several enzymes involved in cholesterol biosynthesis and decrease in expression of Abca1, a transporter regulating cholesterol efflux (86). Consistently, β-glucan administration in mice not only upregulates CD131, a subunit of the receptor for IL-3, IL-5, and GM-CSF, expression in myeloid precursors, but also activates downstream signaling, as demonstrated by STAT5 phosphorylation. The capacity of β-glucan to enhance the biosynthesis of cholesteryl esters and significantly decrease glycerophospholipid-containing arachidonic fatty acid chains highlight the capacity of cells to alter their lipidome and, thus, the physicochemical features of their membranes. This adaptive response has direct consequences in the composition of cellular membranes (87) and consequently in cell signaling (88). In this regard, alterations in the quantitative and qualitative cholesterol composition of the membrane can impact the localization of CD131, its signaling (86, 89) and consequently the differentiation of specific myeloid subsets.

Amino acids, besides being the building block of several molecules, serve as essential precursors of different metabolites. Different studies have shown that glutamine metabolism into glutamate, α-ketoglutarate and succinate semialdehyde can fuel the synthesis of fumarate and succinate for the tricarboxylic acid cycle (TCA) (90). Inhibition of glutaminolysis, in mice, down regulates the production of pro-inflammatory cytokines in monocytes exposed to *C. albicans*, dampening the development of efficient trained monocytes triggered by β-glucan. In line with these observations, the biochemical catheterization of β-glucan trained monocytes has revealed that upon induction of the signaling cascade Dectin-1-Akt-mTOR-HIF-1α, a metabolic shift occurs leading to an increase aerobic glycolysis, glucose usage, lactate production, and TNF-α secretion (67).

Thus, it appears that modulation of metabolic landscape represents a fundamental step to unravel the functional consequences of different monocytes subsets helping to identify new strategies of intervention for the treatment of several patho-physiological conditions. It remains instead undefined whether cancer-derived factors can also generate trained monocytes and if these cells contribute to dampen the anti-tumor response or favor metastatic spread.

## Monocyte Functions

### Monocytes at the Primary Tumor

Tumor derived factors (TDFs) are key mediators in the crosstalk between monocytes and tumor cells. They are involved in monocyte recruitment from the hematopoietic organs in adult life, i.e., bone marrow and in part the spleen, survival, and differentiation within the tumor site. Tumor-released monocyte chemoattractant protein-1 (MCP-1, also known as CCL2) was identified as the major TDF involved in iMo recruitment, through the CCL2-CCR2 axis, into several mouse and human tumors (91). Indeed, inhibition of CCL2-CCR2 signaling in a mouse model of breast cancer significantly impair iMo infiltration and reduce tumor growth and metastases (92). Several studies have described the presence of other chemokines within the tumor microenvironment (TME), including CCL3, CCL4, CCL5, CXCL12, and growth factors such as colony stimulating factor-1 (CSF1), which may also contribute to monocyte recruitment to tumors (93). Indeed, in both mouse (94) and human (91) tumors, cells secrete high level of CSF1 that is involved in recruitment, survival, and differentiation of monocytes. The inhibition of CSF1 signaling in an experimental model of lung carcinoma significantly reduced the number of mature TAMs due to impaired recruitment, proliferation and maturation of iMo cells (95). Moreover, CSF1R signaling blockade can also reprogram immunosuppressive TAMs prompting the differentiation of iMo in anti-tumoral M1-like TAMs (96). However, abrogation of CSF1R signaling, by either small molecules (97) or monoclonal antibodies (98), even though appealing, have so far demonstrated a limited anti-tumor effects. Only a recent work done by Kumar et al. (99) highlighted that the CSF1-dependent cross-talk between tumor cells and cancer-associated fibroblasts (CAF) might explain the inefficacy of such a treatment. Thus, combinatorial therapy targeting both CSF1R and CXCR2 seems to have more chances to generate an effective anti-tumor T cell response. Thus, the therapeutic focus has shifted to combinations of CSF1R inhibitors with other agents. Indeed, treatment with CSF1 inhibitor in combination with either paclitaxel or radiotherapy, is showing to improve the survival of mouse model of breast or prostate cancer, respectively (79, 100) and improve the efficacy of ACT when combined with anti-PD-1 and anti-CTLA4 in a pancreatic mouse model (101). It is becoming clear that cytotoxic therapies, for example, induce mammary epithelial cells to produce monocyte/macrophage recruitment factors, including CSF1 and interleukin-34 (IL-34), which together enhance CSF1R-dependent monocytes/macrophage infiltration, making its inhibition more effective.

One of the strongest stimuli inducing the secretion of TDFs is hypoxia. Indeed, during tumor growth the level of available O_2_ is significantly reduced, especially in the inner part of the neoplastic mass. This hypoxic microenvironment triggers HIF-1α stabilization in tumor cells and the consequent release of pro-angiogenic factors, such as growth factors (VEGF, PDGF, PIGF, ANG-2), chemokines (CXCL8, CXCL12), cytokines (TNFα, IL1β, TGFβ), and metalloproteases (MMPs), which results in the sprouting of new vessels supporting cancer cells growth (102, 103). In addition, hypoxia is a powerful monocyte and macrophage attractant. Through the release of VEGFB and PIGF, tumor cells can enhance haematopoiesis and monocyte recruitment (104). In addition, angiopoietin-2 (ANG-2) is able to recruit circulating Tie2-expressing monocytes (TEMs) that inhibit apoptosis in both tumor and endothelial cells, by mechanisms depending on TNFα release (105), and exhibits an essential pro-angiogenetic role with a not completely clarified mechanism. Interestingly, it was recently discovered that a small subgroup of recruited iMo can be educated by VEGF, and exert their proangiogenic function, supporting the formation of capillaries and larger vessels, as short-lived monocytes without becoming macrophages (106). Secreted VEGF promotes the acquisition of immunosuppressive features in monocytes generating M-MDSCs by upregulating both ARG1 and iNOS through hypoxia response elements and NF-kB (107). In addition, MDSCs can fuel this circuit by releasing MMP-9, which induces VEGF release from ECM (108). Therefore, targeting VEGF/VEGFR has received attention as a strategy to interfere with monocyte-driven angiogenesis. Moreover, blocking this axis will affect monocytes recruitment to the tumor site (109), and favor the conversion from predominant suppressive to anti-tumoral monocytes (110). An additional way to interfere with MDSC differentiation is by interfering with p53 expression, as mentioned before (63).

In the last few years, extracellular vesicles (EVs) emerged among the TDFs as additional determinants in the formation of TME, both at primary and metastatic sites (111). EVs are a heterogeneous group of membrane vesicles mainly composed of exosomes and microvesicles. Interestingly, EVs released by tumor cells (tEVs) and tumor-derived exosomes (TEX) interact with immune cells inducing their switch toward a pro-tumoral phenotype. Particularly, exosomes target monocytes altering their normal function by several mechanisms. In melanoma and colon cancer, TEX block peripheral iMo differentiation into DCs, and favor the acquisition of a peculiar phenotype reminiscent of M-MDSCs and characterized by decreased expression of HLA-DR and costimulatory molecules (112). A similar modulation of monocytes has been described in many other malignancies, including pancreatic cancer, bladder carcinoma, glioblastoma, and multiple myeloma (113–115) and it is often associated with increased cytokine secretion, i.e., CCL2, CCL4, and IL-6, as well as programmed death-ligand 1 (PD-L1) expression (116). Moreover, glioblastoma-derived EVs may skew the differentiation of peripheral blood monocytes to alternatively activated M2 macrophages inducing the expression of elevated levels of VEGF, IL6, Cox2, ARG1, and PD-L1 through STAT3 activation (117). Interestingly, in gastric cancer, TEX effectively educated monocytes to differentiate into a peculiar type of M2 TAM expressing PD-1, which induce T cell dysfunction through IL-10 secretion by interacting directly with PD-L1^+^ cells and thereby promote tumor progression (118). TEX have been described to contain miRNA which can be transferred to target cells and modulate cellular function (119). These data strongly highlight the role of TEX as additional mediators of monocyte dysfunction in TME. In the last few years, significant advances in understanding the mechanisms associated with exosome biogenesis/release have been obtained identifying some possible targets to interfere with this cell-cell communication. Recent studies in mouse models demonstrated that RAB27A or RAB35 inhibition significantly impair TEX secretion in HeLa cervical carcinoma and Oli-Neu oligodendroglial precursor cell lines (120), respectively. Moreover, RAB27A deficient tumor cell lines displayed reduced growth due to impaired recruitment of bone-marrow derived, pro-tumoral immune cells (121).

In addition to be modulated by soluble factors released by the tumor cells, monocytes can shape their effector function also in a contact-dependent manner. For instance, breast cancer stem cells express CD90 and Ephrin A4 receptor (EphA4R) that interact with CD11b and EphA4 present on tumor-associated monocytes and macrophages, respectively, leading to the secretion of inflammatory cytokine (IL-6, IL-8, GM-CSF), which in turn sustain tumor stem cell fate (122).

Among factors that could shape monocyte plasticity, TGFβ, a key multifunctional cytokine, involved in both cancer and inflammation, appears to play a key role. Besides being targeted in a number of human diseases (123), TGFβ has a very well-recognized ability to regulate T cell responses (124), supporting Th9 (125, 126) and Th17 (127) differentiation, and promoting regulatory T cell function (128–130). However, how TGFβ regulates innate immune responses just began to be appreciated. Many TME-associated cells, and among those monocytes and macrophages, express high amount of latent LTGFβ. The recent work by Kelly et al. (131), demonstrates that iMo, beside tumor cells, express high levels of αvβ8 integrin responsible for the activation TGFβ from LTGFβ form (131, 132). Additionally, monocyte-derived macrophages, integrin expression and TGFβ signaling are generally maintained in anti-inflammatory macrophages but down-modulated in pro-inflammatory macrophages. To sustain the immunosuppressive microenvironment, tumor cells exploit this regulatory mechanism upregulating the expression of integrin αvβ8 and activating TGFβ from LTGFβ-expressing monocytes and macrophages (133).

### Monocytes at the Metastatic Niche

Cancer metastasis is a multi-step process of the neoplastic progression termed “invasion-metastasis cascade” (134, 135). Monocytes are, among other myeloid cells (e.g., neutrophils), corrupted to foster tumor progression and metastasis. Accumulating evidence indicates that monocytes (primarily iMo) are essential pre-metastatic promoters being rapidly recruited from the bone marrow to the pre-metastatic niche, mainly by CCL2/CCR2 axis (136, 137), where they promote tumor colonization by secreting angiogenic factors, like VEGFA (92, 138). Indeed, in the MMTV-PyMT mouse model of spontaneous breast cancer, CCL2, released by either tumor cells or stromal cells at the metastatic lung niche, induces the recruitment of CCR2-expressing iMo, which in turn favor the extravasation of tumor cells, through the release of VEGFA (92). Consequently, the inhibition of CCL2-CCR2 signaling axis abrogated the recruitment of monocytes thus reducing metastasis formation. Similarly, in a mouse model of metastatic melanoma (B16F10 model), accumulation of CXCR3^+^monocytes/macrophages in the lung was a prerequisite to mediate melanoma engraftment and metastatic disease (139). However, how this process takes place remains undefined. In a different set of experiments employing B16F10 melanoma model, it was shown that M-MDSCs were recruited to the pre-metastatic niche, mainly by CCL12 expression. By releasing IL-1β, these cells promoted the expression of E-selectin on endothelial cells thus promoting the adhesion of tumor cells to the vascular endothelium (140).

At the metastatic targeted-organs, monocytes can offer survival stimuli for cancer cells. Metastatic cells in the lung, from either mouse or human breast cancer, overexpress vascular cell adhesion molecule-1 (VCAM-1) and shRNA-mediated depletion of VCAM-1 inhibited metastasis formation. Moreover, monocytic cells expressing α4-integrin can bind VCAM-1 present on the surface of tumor cells. Thus, upon α4-integrin engagement on monocytes, VCAM-1 delivers anti-apoptotic signals into breast cancer cells through the PI3K/Akt pathway favoring tumor cell survival (141).

EVs can cross the basal lamina of alveolar capillaries in the lung. In the lungs, alveolar and interstitial macrophages upon taking up these EVs start to secret CCL2 favoring the recruitment of iMo, which in turn differentiate into macrophages, mostly M2-like cells, promoting tumor growth by the secretion of IL-6 and deposition of fibrin (142). Similarly, in colorectal cancer (CRC) patients the high expression of serum exosomal-derived miR-203 was associated with increased probability to develop distant metastases. It was shown that TEX-derived miR-203 uptaken by monocytes promoted their differentiation into M2-like macrophages, *in vitro*. Furthermore, mice injected with CRC cells transfected with miR-203 developed significantly more liver metastases than the control group (143).

We have previously underlined the plasticity of monocytes and how iMo and pMo play different roles in cancer progression and surveillance. In line with these observations, it has been demonstrated that TEX from poorly-metastatic melanoma cells are taken up by bone-marrow monocytes, promoting their differentiation into pMo, which in turn migrate at the metastatic niche clearing tumor cells by direct engulfment or by activating cytotoxic NK cells (144). Interestingly, TEX from poorly-metastatic tumors caused macrophage alteration toward M1-like cells expressing TRAIL, which competed with NK cells for tumor killing. These findings suggest that prior to the acquisition of the metastatic capacity, tumors continuously alert host immune system by producing vesicles that affect innate immune responses and support the concept of developing new cancer immunotherapeutic approach based on TEX to deliver specifically immune triggers.

## Future Prospective: Targeting of Monocytes as the New Frontier for Cancer Immunotherapy

Myeloid cells are extremely plastic and can develop specialized functions in response to micro-environmental pathologic conditions such as infections, autoimmunity or cancer (145). Myeloid cell polarization into either tumor-suppressive or tumor-promoting phenotypes is fundamental for shaping TME. Once at the tumor site, these myeloid cells generally acquire a pro-tumor phenotype (146). Thus, one of the major goals of contemporary tumor immunotherapy is targeting tumor-associated myeloid cells by depletion, recruitment inhibition or reprogramming their polarization/activation status.

As mentioned above, inhibition of the CCL2-CCR2 axis, used to prevent the egression of monocytes from bone marrow, improved the efficacy of chemo-, radio- and immune-therapy in several preclinical models. Nevertheless, the use of either CCL2 or CCR2 inhibitors, in clinical trials, gave disappointing results, indicating the need of supplementary studies considering the presence of potential TME-dependent compensatory mechanisms acting on tumor-resident myeloid cells (146, 147). Moreover, although the continuous blockade of macrophages constrains tumor progression, cessation of the CCL2 blocking therapy stimulates them to a rapid rebound, leading to accelerated metastatic disease via a mechanism dependent on VEGF-A and IL-6 production monocyte-derived by macrophages (102).

### Targeting Trained Immunity

Trained immunity inducing factors were tested for their anti-tumor activity, both *in vitro* and *in vivo*, and some of them reached the clinical application. A β-glucan PAMP, Imprime PGG (Imprime), is currently in clinical development in combination with immune checkpoint inhibitors, tumor-targeting antibodies, and anti-angiogenic antibodies. The results from a randomized phase 2 clinical trial of Imprime in combination with bevacizumab and carboplatin/paclitaxel vs. bevacizumab and chemotherapy alone in the 1st-line treatment of stage IV non-small cell lung cancer showed promising efficacy in terms of both objective tumor response and survival (ClinicalTrials.gov NCT 00874107, EudraCT 2008-006780-37). Earlier results have shown that both the M2 macrophages and DCs derived from Imprime-trained monocytes have higher expression of PD-L1 and CD86, rendering these cells suitable for treatment with anti-PD-1 antibody. *Ex vivo* treatment of T cells with Nivolumab, an anti-PD-1 antibody, enhanced proliferation in response to αCD3/αCD28 stimulation and co-culture with Imprime-trained monocytes-derived M2 macrophages or DCs further improved T cell expansion and increased production of several cytokines, including IFNγ, IL-2, TNF-α, and GM-CSF. Results were further validated in syngeneic mouse model, like the CT26 colon carcinoma. Bacillus Calmette-Guerin (BCG), another trained immunity inducer, is currently the only agent approved by the US Food and Drug Administration for first line treatment of carcinoma *in situ* of the bladder. BCG therapy reduces the risk of recurrence and maintenance therapy with BCG decreases the risk of progression in patients with high-grade, non–muscle invasive bladder cancer (148, 149). It has been speculated that the mechanism of action involves the autophagy. In fact, pharmacologic or genetic inhibition of autophagy blocks the epigenetic reprogramming of monocytes at the level of H3K4 trimethylation, arresting the mechanism of trained immunity induced *in vitro* by BCG. Single nucleotide polymorphisms associated with bladder cancer progression and recurrence, in the autophagy genes ATG2B (rs3759601) and ATG5 (rs2245214), affected both the *in vitro* and *in vivo* training effect of BCG (150).

Muramyl dipeptide (MDP), a synthetic peptide of N-acetyl muramic acid attached to a short amino acid chain of L-Ala-D-isoGln, is a bacterial cell wall peptidoglycan active as NOD2 agonist and contributing to the generation of trained monocytes (151). Interestingly, Paclitaxel conjugated to MDP showed not only antitumor activity, but also immune enhancement capacity. In fact, compared with either paclitaxel or MDP alone, the combination significantly increased the expression and secretion of TNFα and IL-12 from mouse peritoneal monocytes (152). Moreover, it was shown that MDP can upregulate PD-L1 in healthy monocytes, but in patients with Crohn's disease, carrying the Leu1007 frameshift mutation of the NOD2 gene, such effect was completely lost (153) (Table 2).

**Table 2:** Inhibitors and their corresponding targets found to impact pathways regulating different aspects of monocyte biology.

**Effect**	**Target**	**Drug name**	**Targeted monocyte subset**	**References (PMID)**
Recruitment abrogation	CCR2	PF04136309 Carlumab	Inflammatory monocytes M-MDSCs	27055731 22907596
	CFS1R	ARRY-382 FPA008 GW2580	Patrolling monocytes M-MDSCs	29872489 20008303 16249345
	IL-6R	mAb 15A7	Patrolling monocytes M-MDSCs	28235765 22653638
	Attenuating RNS generation	AT38	M-MDSCs	21930770
	Multi-kinase	carbozantinib BEZ235	M-MDSCs	28321130
	amino-biphosphonates	matrix metalloproteases	M-MDSCs	12912933
Apoptosis induction	FLIP	5-fluorouracil Gemcitabine Docetaxel Paclitaxel Oxaliplatin Cisplatin Irinotecan Etoposide	M-MDSCs	30518925
	Fas	IL-2 with anti-CD40 antibody (clone FGK115B3)	M-MDSCs	24808361
	IL1R	Anakinra	Monocytes	29808007
Inhibition of proliferation	GM-CSF	mAb clone MP1-22E9	M-MDSCs	22698406
	G-CSF	mAb clone MAB414	M-MDSCs	19346489
	VEGF	mAb clone G6.23	M-MDSCs	17664940
Metabolic alteration	Mevalonate-cholesterol pathway	Statins	Trained monocytes	29328908
	NOD2	Muramyl dipeptide	Trained monocytes	
	mTOR	Everolimus Metformin	Trained monocytes	25258083 27926861 23415113
	Bromodomains	I-BET151	Trained monocytes	27863248
	Histone deacetylase	JIB-04	Trained monocytes	23792809 29702467
	Glutamine-pathway	DON	Trained monocytes	30541099 297024
Immunosuppressive function	pSTAT3	Stattic	M-MDSCs	23454751
	COX2	Celecoxib	M-MDSCs	21324923
	IDO1	1-methyl-L-tryptophan Epacadostat	M-MDSCs	23440412
	ARG1	CB-1158 NCX 4016 NG-hydroxy-L-arginine [NOHA] Nω-hydroxy-nor-Arginine [Nor-NOHA]	M-MDSCs	29254508 29133913
	Phosphodiesterase (PDE5)	Sildenafil, tadalafil	M-MDSCs	27495172 25564570
	PD-L1/CTLA-4	Atezolizumab ipilimumab	M-MDSCs	28364000 30267200
Cell differentiation	Retinoic acid receptor	ATRA	M-MDSCs	18006848
	ENTPD2	POM-1	M-MDSCs	28894087

### Targeting Signaling Pathways

An alternative approach to target trained immunity is to inhibit the pathway Dectin-1-Akt-mTOR-HIF-1α. To this end, the beneficial effects of Metformin and Everolimus, an mTOR activator rapamycin analog, administration to patients with type 2 diabetes and cancer, respectively, were linked with the modulation of trained monocytes (67, 90, 154). Interestingly, inhibitors of other kinases, such as Raf-1, PI3K, and ERK are of particular interest in modulating trained monocytes because they represent downstream effectors of Dectin-1 and NOD2 activation (Table 2). In particular the knockout of PI3Kγ was reported to break tumor tolerance by MDSC reduction (54) as well as, the combination targeting of PI3Kδ in association with PD-L1- based immunotherapy better limited tumor progression (155, 156). Moreover, the pharmacological treatment using multi-kinase inhibitors carbozantinib and BEZ235, which limit MDSC accumulation, in combination with immune checkpoint therapy controlled more efficiently tumor growth in a castration-resistant prostate tumor model than the single agents (157) underlying the possibility to overcame *de novo* resistance to antibody blockade based therapy by limiting MDSCs.

The development of epigenetic modulators is acquiring increased interest due to the relevance of epigenetic changed in several diseases. The broad jumonji histone demethylase inhibitor JIB-04 decreased trained immunity response by modulating of the methylation status of H3K9 (158). Interestingly, a clinically relevant small molecule of the BET family of bromodomains, I-BET151, was shown to prevent monocyte tolerance when administered concomitantly with LPS, but it was ineffective when administered after LPS stimulation. These results suggest that I-BET151 is not an effective treatment in monocytes that have already experienced an inflammatory response (159) (Table 2).

### Targeting Metabolic Pathways

Metabolic modulation also represents another interesting approach to target both trained monocytes and M-MDSCs. Some metabolites and metabolic enzymes function as either substrates or cofactors for chromatin modifying enzymes, thereby influencing the epigenetic landscape of target cells. Accordingly, fumarate can increase trained immunity by increasing H3K4me3 and H3K27ac and inhibiting the degradation of HIF-1α (90). Moreover, the decreased expression of lysine demethylase 5 family of HDAC (KDM5), responsible for H3K4 demethylation, is also inhibited by fumarate, maintaining then the accessibility of the chromatin. On the other hand, it was shown that tolerant monocytes lack the activity of KDM5, whose function can be restored by its cofactor, α-ketoglutarate (90). Mevalonate, intermediate of the cholesterol pathway has been shown to induce trained immunity (74), consequently, statins can be used to prevent this process under conditions in which accumulation of trained monocytes is detrimental, like patient with hyper-IgD syndrome or inflammatory conditions. Additionally, given the enhanced glutamynolysis associated to trained immunity (90), administration of 6-diazo-5-oxo-L-norleucine (DON), which inhibits glutamine uptake and metabolism, was shown to have favorable effects after organ transplant preventing rejection (160) (Table 2). Even though targeting metabolic pathways, to modulate trained monocyte function, is feasible, toxicity, and side effects represent the main drawbacks of metabolic drugs. Overcoming this limitation, for example, by delivering drugs trough nanoparticles/nanocarriers, could open the access to a variety of molecules that have already demonstrated their efficacy *in vitro*.

### Targeting Immunosuppressive Monocytes (M-MDSC)

Together with trained monocytes, MDSCs represent the other group of targetable cells. Proliferating T cells need a large supply of amino acid like L-arginine and L-tryptophan. MDSCs have developed a strategy to modulate local concentrations of these amino acids via the up regulation of enzymes involved in their degradation like ARG1, NOS2, and IDO. Developing inhibitors of these enzymes represent a field of intense research. To this end, nitroaspirin, consisting of a nitric oxide group covalently linked to aspirin, was shown to restore L-arginine levels in T cells, by suppressing the production of ROS and iNOS (161). Moreover, our laboratory showed that treatment with AT38 [3-(aminocarbonyl) furoxan-4-yl]methyl salicylate], decreased MDSC-induced nitration within the tumor environment, increasing CCL2 binding and T cell tumor infiltration in mice (60). N-hydroxy-L-arginine (NOHA) is an intermediate in the conversion of arginine to citrulline and NO by iNOS (162) (Table 2). It is a potent physiologic inhibitor of ARG1. Mice exposed to NOHA demonstrated inhibition of MDSC function, and mice with B cell lymphoma treated with NOHA had decreased numbers of circulating Treg cells and improved immune responses to the cancer (163). We also demonstrated that the production of polyamine by ARG1 activity promotes IDO1 activation through Src kinase signaling (164). Therefore, a combined targeting of ARG1 and IDO1 using pharmacological compounds (165) could be an effective treatment to constrain tumor-associated immunosuppression improving cancer immunotherapy. MDSCs generate an immunosuppressive environment also by producing prostaglandin E2 (PGE2), which levels are regulated through the enzyme Cox2 (166). PGE2 activates PGE2-R on MDSCs altering the differentiation of MDSCs. In the bone marrow, activation of PGE2-R hampers the differentiation of monocytes into antigen presenting cells, while increased PGE2 switch monocytes into MDSCs via increased expression of IDO, IL-4Rα, iNOS, and IL-10 (167, 168). In line with these findings, blocking the production of PGE2 in mice bearing lung carcinoma, with Cox2 inhibitors, decreased the expression of ARG1 in MDSC and tumor growth (169). Cox2 inhibitors may also provide other antitumor effects (Table 2). Celecoxib, a Cox2 inhibitors, was shown to decrease MDSC recruitment and increased CD8^+^ T cell tumor infiltration in gliomas and colon carcinoma by decreasing CCL2 production (170).

Methionine, an essential amino acid for normal T cells function, is generally supplied by antigen presenting cells. DCs and macrophages import cysteine to create methionine, which is then secreted; they additionally release thioredoxin converting cysteine to methionine. However, in the TME, MDSCs transport cysteine intracellularly thus depleting T cells of methionine (171, 172). Consequently, blocking thioredoxin could prevent T cells proliferation arrest. In this situation small molecules or neutralizing antibodies targeting extracellularly released enzymes could be beneficial to restore T cells function and used in combination with ACT of tumor specific T cells or with check point inhibitors.

Constitutive activation of the JAK-STAT pathway has been implicated in the proliferation of MDSCs via anti-apoptotic and pro-proliferative genes (173). Moreover, ARG1 and iNOS, immunosuppressive enzymes in MDSCs, are controlled via STAT1 and STAT3 (174). Consequently, inhibition of the JAK-STAT pathway has been of great interest. Many inhibitors of STAT1/STAT3 have been discovered and several of them already enter clinical trials. Among those one, Stattic, an inhibitor of pSTAT3, reduces the suppressive activity of MDSC *in vitro* both in mice and in humans (175, 176) (Table 2).

In the last 10 years, several approaches to target immunosuppressive monocyte referred as M-MDSC were developed using a large spectrum of pharmacological compounds and immunotherapeutic approaches with the aim to limit MDSC proliferation, function, and recruitment to tumor site. For instance, non-therapeutic low doses of conventional chemotherapeutic drugs such as gemcitabine and 5-fluorouracil [which does not induce immunogenic cancer-cell death of tumor cells (177)] were able to limit Ly6C^+^MDSC number and activity by inducing c-FLIP down-regulation (65). The M-MDSC elimination is essential to restore the immune response in tumor-bearing mice by rescuing the frequency of circulating anti-tumor T cells (51). These data obtained in preclinical cancer models were also confirmed in tumor patients. In fact, renal cell cancer (RCC) patients with low frequency of M-MDSCs showed a better disease control after anti-tumor peptide-based vaccination in combination with chemotherapy (178); moreover, the HPV16 long-peptide-based vaccination during CarboTaxol treatment was able to reduce circulating MDSCs and generated a strong immune response, confirming the relevance of M-MDSC elimination as a valid approach to enforce anti-tumor immune response (179). Based on these data, several chemotherapeutic compounds with different mechanisms of action were listed as M-MDSC-targeting drugs (173). M-MDSC elimination can be also achieved using immune compounds such cytokines and antibodies. In this line, the combination of IL-2 with anti-CD40 antibody was effective on cancer growth control in two different mouse tumor models by inducing M-MDSC elimination trough Fas-mediated apoptosis (180). Similarly, to improve the efficacy of CAR T cells immunotherapy in leukemia setting, by limiting transferred T cells-associated life-threatening cytokine-release syndrome (CRS) and neurotoxicity, either monocyte-depletion or infusion of IL-1 receptor antagonist anakinra were reported as effective strategies (181). Other therapeutic approaches aim at favoring MDSC differentiation into anti-tumoral cell subsets such as macrophages and DCs. For example, all-trans-retinoic acid (ATRA) treatment was reported to promote the differentiation of MDSC into mature anti-tumoral myeloid cells via the activation of the ERK1/2 signaling pathway (182) as well as the treatment with 25-hydroxyvitamin D3 was reported to reduce the frequency of immature immune suppressive cells in peripheral blood of HNSCC patients (183). Moreover, M-MDSC differentiation toward mature anti-tumoral monocytes-derived DCs can be achieved using pharmacological inhibitor of ATP-converting ectoenzyme ENTPD2, thus mitigating cancer growth and enhancing the efficiency of immune checkpoint inhibitors (184). Finally, some targeting approaches aim at blocking M-MDSC migration from bone marrow to tumor site. For example, amino-biphosphonates were able to prevent the activation of matrix metalloproteases (MMPs) limiting cancer aggressiveness and distal spread (185). Along the same line, pharmacological antagonist of chemokine receptors (i.e., S-265610, CXCR2-specific antagonist) were able to drastically reduce M-MDSC in tumor-bearing mice (186).

## Concluding Remarks

Monocytes are relevant immunological cells connecting the innate and adaptive immune compartments. Here, we have attempted to integrate recent advances in the molecular, metabolic, and functional aspects of monocyte biology with the current state of understanding about the role of these cells in cancer growth and metastatic spread. Although a large body of evidence supports the notion that circulating monocytes serve only as precursor cells that replenish tissue macrophages and DC populations, the overwhelming complexity underlined by high throughput technologies, is supporting a direct contribution of monocyte to cancer development. Targeting monocytes at the immunological, metabolic, epigenetic, and transcriptional level is a promising strategy to treat both disease with impaired immune function, like cancer, or with over-reactive immune response, like autoimmune diseases. The advances in our knowledge on monocyte development, response, and reprogramming, particularly during cancer evolution and metastatic spread, will pave the way for the development of new therapeutic strategy with specificity and limited toxicity.

## Author Contributions

SC, SU, RT, IM, FD, and SS wrote parts of the manuscript. SC and VB edited and finalized the manuscript.

### Conflict of Interest Statement

The authors declare that the research was conducted in the absence of any commercial or financial relationships that could be construed as a potential conflict of interest.
